# Editorial: Research priorities concerning formal and informal learning in low- and middle-income countries

**DOI:** 10.3389/fpsyg.2024.1391089

**Published:** 2024-03-19

**Authors:** Julie Ann Robinson

**Affiliations:** College of Education, Psychology and Social Work, Flinders University, Adelaide, SA, Australia

**Keywords:** learning, education, publication bias, low- and middle-income countries, Africa, WEIRD contexts

Editorials typically conclude by situating the Research Topic within a wider context. However, the aims of the current Research Topic can be best explained by providing this context at the outset. During the past 20 years, researchers and practitioners in education, psychology, and health disciplines based in high-income countries have increasingly reflected on the limitations of their knowledge base (Arnett, 2008; Begeny et al., 2018; Cash-Gibson et al., 2018; Thalmayer et al., 2021; Molina-Aguilar and Robles-Espinoza, 2023). Most published research in these disciplines continues to be conducted with the small segment of the global population that lives in North America, Western Europe, and high-income countries in the Asia-Pacific region. These participants have life experiences that are not representative of the majority (Henrich et al., 2010). Research from the USA shows the greatest over-representation (Table 1a). The label “WEIRD” (Western, Educated, Industrial, Rich, and Democratic; Henrich et al., 2010) aptly captures the atypicality of the contexts in which most published research has been conducted.

**Table 1:** Summary of results from selected studies of the over-representation of (a) samples and authors and (b) editors from the USA, and North America and Europe in research journals related to education, psychology, and health.

**(a) Samples and authors**				**% samples**			**% first author**	
**Field and authors**	**Journals**	**Years**	**Articles**	**USA**	**All WEIRD countries** ^∧^		**USA**	**All WEIRD countries** ^∧^
**Psychology: General**								
Arnett (2008)	6 APA journals	2003–2007	4,037	68%	96%		72%	100%
Thalmayer et al. (2021)	6 APA journals	2014–2018	3,447	67%	96%		64%	100%
Rad et al. (2018): Study 1	*Psychological Science*	2014	428	50.8%	82.8%		-	-
Rad et al. (2018): Study 2	*Psychological Science*	2017	40	51.1%	74.1%		-	-
**Psychology: Educational**								
Begeny et al. (2018)	8 specialist journals	2002–2016	4,456	45.3%	90.2%^*^		55.0%	94.0%^*^
**Developmental Science**								
Nielsen et al. (2017)	3 highest impact journals	2006–2010	1,582	57.7%	90.5%^*^		60.1%	95.9%^*^
Moriguchi (2022)	*Infant and Child Development*	2006–2020	152	35.5%	88.8%^*^		93.0%	36.1%^*^
Singh et al. (2023)	4 specialist journals	2011–2022	968	NA	87%^*^		NA	NA
**Health**								
Cash-Gibson et al. (2018)	Articles on health inequities	1966–2015	29,379	NA	NA		48.5%^#^	92.7%^#^
**(b) Authors and editors**			**% authors**			**% editors**		
**Field and authors**	**Journals**	**Years**	***n***	**USA**	**N. America and Europe**	***n***	**USA**	**N. America and Europe**
**Psychology: General**								
Lin and Li (2023)	68 journals in 10 fields	2017–2019	37,801	44.1%	85.9%	5,653	60.3%	91.4%
Palser et al. (2022)	50 English-language journals	Editors: 2019	NA	45.4%	89.2%	2,864	60.6%	94.3%
		Authors:2014–18^+^						
**Psychology: Educational**								
Wang et al. (2020)	45 specialist journals	2017	NA	NA	NA	1,605	54%	80%^~^
**Neuroscience**								
Palser et al. (2022)	50 English-language journals	Editors: 2019	NA	35.5%	84.7%	3,093	52.4%	86.3%
		Authors:2014–18^+^						
**Health**								
Melhem et al. (2022)	5 leading journals in 9 fields	2020	10,096	NA	73.6%	3,819	NA	88.4%

APA, American Psychological Association; NA, Not available.

∧Western, educated, industrialized, rich, and democratic populations (Western Europe, USA, Canada, other English-speaking countries including UK, Australia, New Zealand, and Israel).

^*^Excludes Israel.

^#^All authors.

^+^Last author on article.

^~^Includes Australia and New Zealand.

Neither the location of high-impact journals (e.g., Palser et al., 2022) nor the geographic distribution of professionals in relevant fields (e.g., Begeny et al., 2018) explain this bias. One contributor appears to be the over-representation of editors in North America and Europe compared to the geographic distribution of the authors of articles in their journals (Table 1b). Reviewers have also been implicated (Harris et al., 2017; Aly et al., 2023). Because editors and reviewers influence what is published, they can influence not only the career opportunities of individual researchers but also the direction of a field of research.

Van de Vijver (2013) provides three reasons for broadening the evidence base: we could learn how to tailor, expand and apply discipline knowledge to benefit the majority of the world's population; we could test implicit assumptions about the global validity of findings and theories originating in WEIRD contexts; and more inclusive research would allow more appropriate evidence-informed practice by policy-makers and professionals in education, psychology, and public health.

Increased awareness of the limited scope of published research and its negative consequences has not led to greater inclusivity (Table 1a). Researchers from WEIRD contexts who are interested in conducting research in the majority world face the challenges of building trust relationships in another cultural setting, the need for additional funding, and complex ethical issues (Hyder et al., 2012; Smith et al., 2014; Regmi et al., 2017; Morelli et al., 2018; Lansford et al., 2019; Steinert et al., 2021; Raval et al., 2023). Moreover, simply transplanting research from WEIRD to majority world contexts can only partially achieve the goals of increased inclusivity. Because culture and environment influence the questions researchers ask (Rad et al., 2018; Lin and Li, 2023), “a diverse science must include a diverse group of scientists” (Rad et al., 2018, p. 11,404). Unfortunately, researchers in low- and middle-income countries also face numerous barriers to contributing to the diversity of published research: they may have access to electricity for only limited hours each day; governments may limit or prevent access to the internet and email; institutions may be unable to provide journals or academic books, data analysis tools, or rigorous training in methodologies and reporting conventions favored by high-impact journals (Nielsen et al., 2017; Draper et al., 2023). In addition, research conducted in low- and middle-income countries may be informed by epistemologies, methods, and analyses that are rigorous but unfamiliar to reviewers and editors in high impact journals.

The current Research Topic sought to make a small but meaningful contribution toward greater inclusivity in published research. It had four distinctive features. First, it shifted the focus to research priorities in the majority world, which may be overlooked, take different forms, or hold different significance in research conducted in WEIRD contexts. Second, to empower researchers from low- and middle-income countries, all manuscripts were required to include at least one author from these countries. Twenty-nine manuscripts were submitted. Twelve of the 14 accepted manuscripts had a first author from a low- or middle-income country, and nine had only authors from these countries. The samples were drawn from nine countries across South America (Brazil), Asia (Thailand, Vietnam), and Africa (Cameroon, Cote d'Ivoire, Kenya, Nigeria, Uganda, Zimbabwe). In addition, one article focused on the sub-Saharan African region, while another had a global scope. The third and fourth distinctive features of the Research Topic were the result of a unique collaboration with *Frontiers in* journals. The Research Topic was accepted as a developmental project. This allowed editors to offer additional support and the opportunity to make multiple revisions when it was perceived that authors could benefit from this. While such openness increased the number and diversity of manuscripts that were accepted for publication, it came at a cost. Due to the very extended timeline, each of my co-editors needed to step down from their roles due to competing commitments or ill-health. However, the success of the project would not have been possible without the contributions of Suman Verma (Panjab University, India), Peter Kakubeire Baguma (Makerere University, Uganda), and Josafa Moreira da Cunha (Universidade Federal do Paraná, Brazil). In addition, *Frontiers in* journals awarded the Research Topic “Global Challenge” status, which provided a grant that covered most of the article processing fees for authors who did not have other sources of funding. This was essential since, in one case, the article processing fee represented more than 25% of the first author's gross annual income.

The Research Topic focused on formal and informal learning because these are key mechanisms for achieving many national development goals, they expand the life opportunities of individuals and equip them to make a wider range of contributions to their communities. Learning, broadly defined, is relevant to many academic disciplines. The 14 accepted manuscripts were published across four journals (*Frontiers in Education, Frontiers in Psychology, Frontiers in Public Health*, and *Frontiers in Environmental Science*).

The diverse domains of learning addressed by the accepted manuscripts are summarized in Figure 1. Most of the topics addressed within these domains fell into three broad categories. The first category dealt with the relationship between education and issues that are most prevalent in low- and middle-income countries: stunting (Brou et al.; Robinson and Dinh), adolescent pregnancy (Opiyo and Elizabeth), persistent collective violence (Shockden et al.), and protracted and complex refugee/migration contexts (Pooseesod et al.). In low resource settings, many education systems offer insufficient places to meet demand, and lack the basic infrastructure, adequately trained teachers, and teaching and learning resources necessary to support effective learning. The second category of manuscripts explored the mobilization of community resources as a strategy to mitigate these limitations in early childhood education (Tafirenyika et al.), schools (Balikoowa et al.), and vocational training (Ngyah-Etchutambe et al.). The third category of manuscripts explored formal and informal learning contexts that draw on distinctive cultural objectives, resources, and pedagogical practices reflected in culturally informed preschool programs (Dinh and Robinson), folktales (Wiysahnyuy and Valentine), and patterns of child rearing (Tchombe). The final three manuscripts examined the role of education and financial inclusion in poverty reduction across 68 low- and lower-middle-income countries (Shi and Qamruzzaman), the association between race-based victimization and academic functioning in a nation-wide sample of Brazilian secondary school students (da Cunha and Santo), and a training program to reduce violence directed toward Vietnamese women and children (Yount et al.).

**Figure 1:**
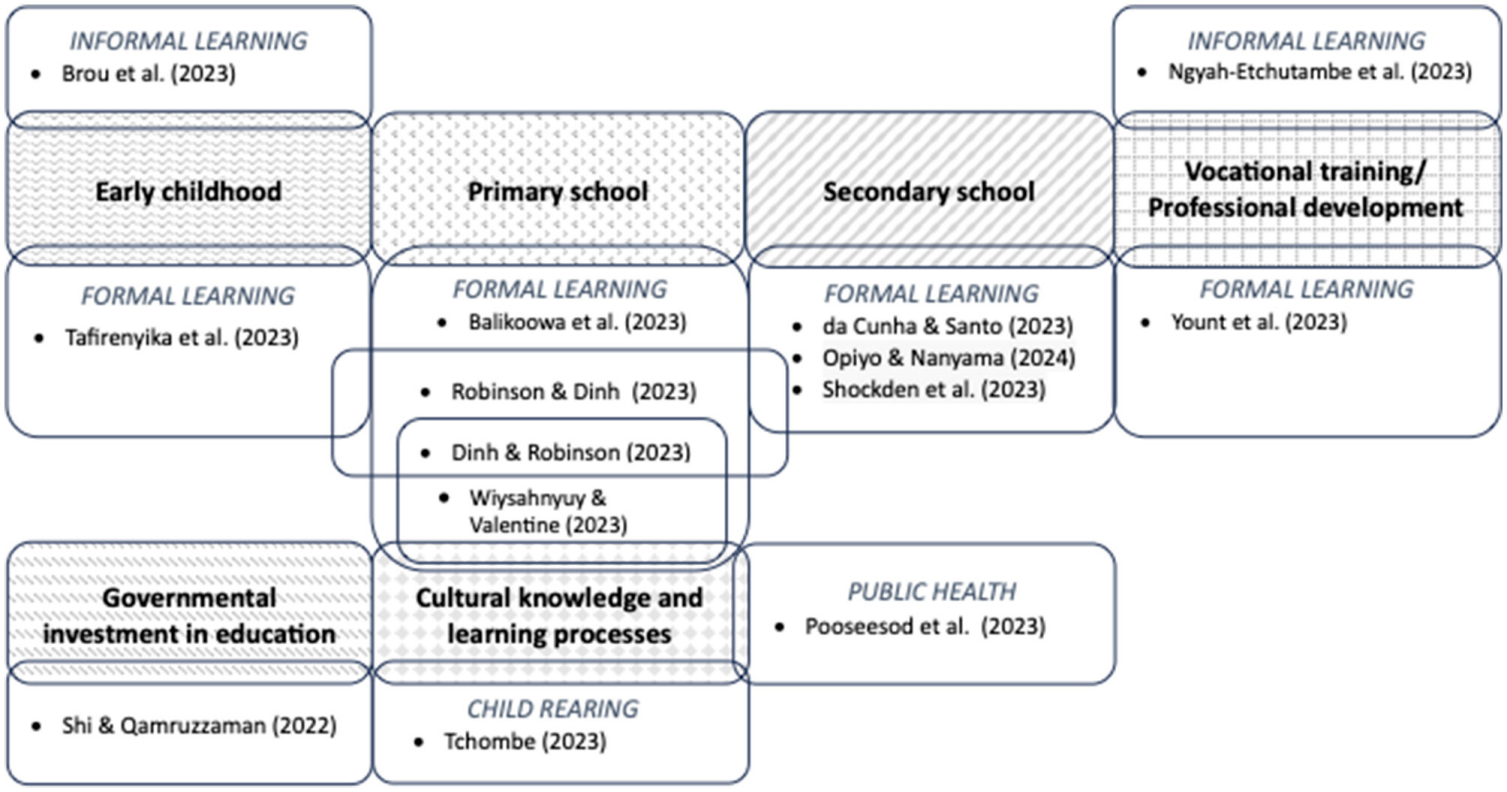
Diversity in accepted manuscripts in the Research Topic “*Research priorities concerning formal and informal learning in low- and middle-income countries*.”

In conclusion, this Research Topic demonstrates one way in which authors, reviewers, editors, and publishers can collaborate to remove many of the barriers that can prevent researchers from low- and middle-income countries from achieving publications in high impact journals. Publications in open access journals are particularly important since these are often the only source of research available to other scholars in low- and middle-income countries. The 14 articles provide insights into the diversity of issues, research questions, samples, and findings that can be achieved by a more inclusive approach to the publication of research.

## Author contributions

JR: Writing – original draft, Writing – review & editing.
